# Identifying Ventricular Dysfunction Indicators in Electrocardiograms via Artificial Intelligence-Driven Analysis

**DOI:** 10.3390/bioengineering11111069

**Published:** 2024-10-26

**Authors:** Hisaki Makimoto, Takayuki Okatani, Masanori Suganuma, Tomoyuki Kabutoya, Takahide Kohro, Yukiko Agata, Yukiyo Ogata, Kenji Harada, Redi Llubani, Alexandru Bejinariu, Obaida R. Rana, Asuka Makimoto, Elisabetha Gharib, Anita Meissner, Malte Kelm, Kazuomi Kario

**Affiliations:** 1 Cardiovascular Centre, Jichi Medical University, Shimotsuke 329-0498, Japan; kabu@jichi.ac.jp (T.K.); takahide.kohro@jichi.ac.jp (T.K.); y-ogata@jichi.ac.jp (Y.O.); haradak@jichi.ac.jp (K.H.); kkario@jichi.ac.jp (K.K.); 2 Graduate School of Information Sciences, Tohoku University, Sendai 980-8577, Japan; takayuki.okatani.a2@tohoku.ac.jp (T.O.); masanori.suganuma.b4@tohoku.ac.jp (M.S.); 3 Data Science Centre, Jichi Medical University, Shimotsuke 329-0431, Japan; 4 Division of Cardiology, Pulmonology and Vascular Medicine, Medical Faculty, Heinrich-Heine University, 40225 Düsseldorf, Germany; redillubani@yahoo.com (R.L.); alexandru.bejinariu@med.uni-duesseldorf.de (A.B.); obaida.rana@med.uni-duesseldorf.de (O.R.R.); malte.kelm@med.uni-duesseldorf.de (M.K.); 5 Clinical Research Center, Jichi Medical University, Shimotsuke 329-0498, Japan; 6 CARID, Cardiovascular Research Institute Düsseldorf, Medical Faculty, University Hospital Düsseldorf, Heinrich-Heine-University, 40225 Düsseldorf, Germany

**Keywords:** electrocardiogram, ventricular dysfunction, artificial intelligence

## Abstract

Recent studies highlight artificial intelligence’s ability to identify ventricular dysfunction via electrocardiograms (ECGs); however, specific indicative waveforms remain unclear. This study analysed ECG and echocardiography data from 17,422 cases in Japan and Germany. We developed 10-layer convolutional neural networks to detect left ventricular ejection fractions below 50%, using four-fold cross-validation. Model performance, evaluated among different ECG configurations (3 s strips, single-beat, and two-beat overlay) and segments (PQRST, QRST, P, QRS, and PQRS), showed two-beat ECGs performed best, followed by single-beat models, surpassing 3 s models in both internal and external validations. Single-beat models revealed limb leads, particularly I and aVR, as most indicative of dysfunction. An analysis indicated segments from QRS to T-wave were most revealing, with P segments enhancing model performance. This study confirmed that dual-beat ECGs enabled the most precise ventricular function classification, and segments from the P- to T-wave in ECGs were more effective for assessing ventricular dysfunction, with leads I and aVR offering higher diagnostic utility.

## 1. Introduction

Despite advancements in medical technology, sudden cardiac death (SCD) remains a leading cause of mortality worldwide. Patients with heart failure, particularly those with ventricular dysfunction, are at an increased risk of SCD, making early detection crucial for identifying high-risk cases.

Currently, the decline in ventricular function is diagnosed through cardiac imaging tests in clinical medicine [1,2], and diagnosing cardiac function from an electrocardiogram (ECG) has been not feasible. However, previous research indicates that artificial intelligence (AI) technologies can accurately determine cardiac dysfunction from ECGs, offering a promising tool for early screening and intervention [3,4].

Nevertheless, the basis on which AI identifies ventricular dysfunction from ECGs is not clearly understood. Identifying the signals indicating ventricular dysfunction within ECGs would streamline clinicians’ work. Thus, this study aimed to identify the localisation of ECG signals that AI uses to determine ventricular dysfunction.

To address this, the following clinical questions were formulated: (1) Can multiple heartbeats and/or intervals between them on an ECG help detect ventricular dysfunction? (2) Does the presence of signals capable of detecting ventricular dysfunction vary across different ECG leads? (3) Which specific ECG segment within a single heartbeat contains the most pronounced signals for detecting ventricular dysfunction? This study aimed to resolve these questions by analysing the indicated aspects of ECG data for potential signs of ventricular impairment.

## 2. Methods

### 2.1. Dataset Preparation

This study was approved by the Institutional Review Boards of Jichi Medical University (JMU, Tochigi, Japan) (RINFU23-093) and University Hospital Düsseldorf (UHD, Düsseldorf, Germany) (study number: 2019-763). Inclusion criteria included (1) age ≥ 18 years, (2) availability of a complete set of echocardiography tests, and (3) availability of digital 12-lead ECG recordings within 1 month of echocardiography.

At JMU Hospital in Japan, a dataset of 17,021 ECGs (10,626 unique cases, 12 leads, 500 Hz, 10 s) with corresponding echocardiography results between 2014 and 2019 was compiled for training and internal validation. Additionally, 401 unique cases from UHD in Germany, collected in 2021, were prepared for external validation.

ECGs were recorded as digital data at 500 Hz for 10 s. Digital filters were set according to institutional provisions and examiner protocols. At JMU, the FCP-8700 (Fukuda Denshi, Tokyo, Japan) was used, whereas MAC1200 or MAC 2000 (GE HealthCare, Chicago, IL, USA) was used at UHD. The digital ECG data were exported as CSV files, each containing 12 rows and 5000 columns.

All echocardiography examinations were performed in accordance with guidelines from the Japanese Circulation Society and the European Association of Cardiovascular Imaging (EACVI) in our respective echocardiography laboratories at JMU and UHD [5,6].

The cases were categorised based on echocardiography results into two groups, namely normal left ventricular function and decreased left ventricular function (<50% ejection fraction). Within each group, ECGs were randomly divided into five subsets, with four used for training and development and one for internal validation (Table 1). Cases with decreased ventricular function were further categorised into mildly reduced ejection fraction (mrEF: 40% ≤ left ventricular ejection fraction [LVEF] < 50%) and reduced EF (rEF: LVEF < 40%), and each subgroup underwent specific preprocessing steps [2].

We employed a modified stratified four-fold cross-validation approach using the four subsets for model development (Supplementary Materials). The JMU ECG dataset was stratified according to LVEF to ensure an equal distribution of cases with reduced LVEF across the four independent training and development groups, with no overlap. Test cases remained consistent across all folds to prevent overlap with training data.

The UHD ECG dataset served as an external validation set. We balanced the number of cases with and without reduced LVEF arbitrarily, aiming to adequately evaluate the sensitivity of the developed models.

### 2.2. Data Preprocessing

**Figure 1:**
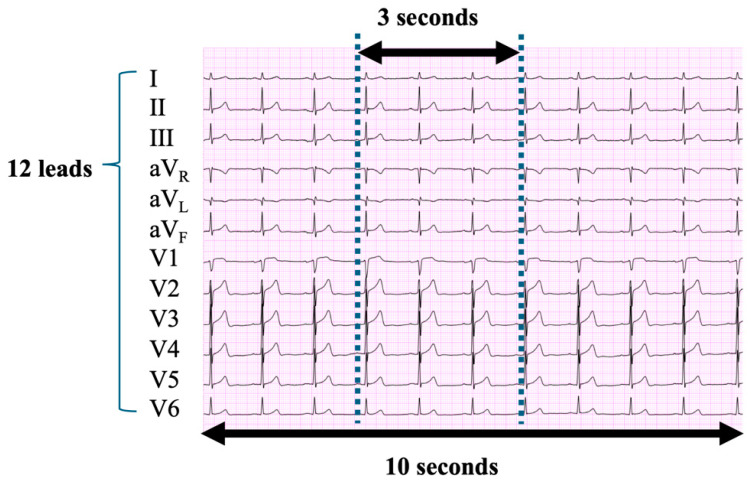
Extraction of 3 s ECG strips. We extracted 3 s, 12-lead ECG strips from the original 10 s ECG recordings. Multiple 3 s intervals were obtained as part of an oversampling strategy, determined by predefined time steps based on the left ventricular ejection fraction. See the main text for further details. ECG, electrocardiogram.

**Figure 2:**
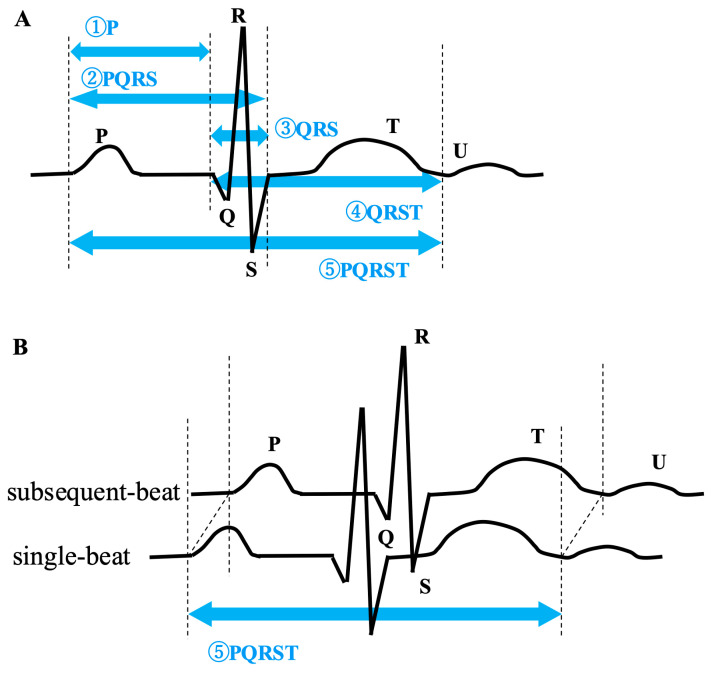
Beat-wise ECGs and partial-beat ECGs. (**A**) Each beat-wise ECG underwent segmentation, and the segmented ECG data were extracted. Five different segmentations were conducted: ① P segment, ② PQRS segment, ③ QRS segment, ④ QRST segment, and ⑤ PQRST segment. (**B**) Two-beat ECGs were constructed by combining the subsequent PQRST complex channel-wise. ECG, electrocardiogram.

For preprocessing in AI model construction, the collected dataset was standardised as follows:

(1) Three-second ECG segmentation: Each record was segmented into multiple 3 s intervals as part of an oversampling strategy based on predefined time steps aligned with the LVEF (Figure 1). In the training datasets, the oversampling factors were x4 for preserved EF (pEF), x7 for mrEF, and x6 for rEF. For the development and test datasets, the oversampling factors were x4 for pEF, x16 for mrEF, and x11 for rEF. The segmented 3 s ECG data were then exported as CSV files, each containing 12 rows and 1500 columns.
(2) Single-beat ECG extraction: Using Ngaia™ from Nagaoka Industries (Nishinomiya, Japan), ECG data were segmented based on the detected QRS complex. Data spanning 250 ms before and 500 ms after the QRS complex were extracted. The data extended to the end of the T-wave and were replaced with the value at the end of the T-wave. These segmented single-beat ECG data were then exported as CSV files, each containing 12 rows and 375 columns.

(3) Partial ECG: From a single-beat ECG, five types of partial ECGs were created, demarcated by the data points 100 ms before the QRS, at the ST junction, and at the end of the T-wave, including ① P, ② PQRS, ③ QRS, ④ QRST, ⑤ PQRST (Figure 2). Based on the single-beat ECG data, the partial ECGs were segmented as follows: the boundary between the P-wave and QRS complex was defined as 100 ms before the detected QRS complex. The end of the QRS complex and the T-wave was also detected using the Ngaia™ programme.
(4) Two-beat ECG composition: Using the same Ngaia™ software, two-beat ECGs were constructed by combining subsequent QRS complex channel-wise. This resulted in a dataset with 24 channels composed of 24 rows and 375 columns in CSV files. The last QRS complex of a 10 s ECG, lacking a subsequent QRS complex, was combined with the first QRS complex of the same interval.
(5) Reduced lead ECG: ECGs recorded in 12 leads were divided into individual lead data. Selected rows from the single-beat ECG data were utilised, resulting in datasets comprising between 1 and 12 rows and 375 columns. The total number of lead combinations was 40,955 (12C1 + 12C2 + … + 12C12).

Data standardisation was performed immediately before model training using the overall mean and standard deviation of the training dataset. The development, internal, and external validation datasets were standardised using the same mean and standard deviation values applied to the training dataset.

### 2.3. Architecture of AI

**Figure 3:**
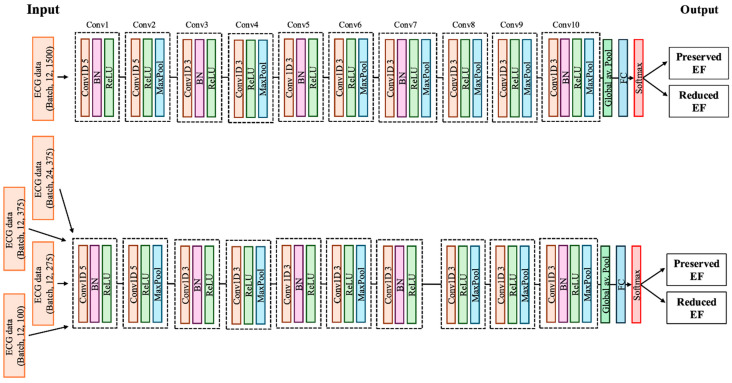
The architecture of the convolutional neural network. ECG, electrocardiogram; EF, ejection fraction.

The convolutional neural network (CNN) architecture used in this study is illustrated in Figure 3. The model developed for analysing single-beat ECGs was a one-dimensional CNN comprising multiple blocks. Each block consisted of a one-dimensional convolution, batch normalisation, a ReLU activation function, and a max pooling operation. The last layer included a fully connected layer and softmax function to estimate the probabilities of the following two diagnostic categories: pEF and reduced LVEF. The detailed explanation on the model architecture was given in the Supplementary Materials. For the model analysing double-beat ECGs, the same CNN architecture was employed with an increased number of input channels. All analyses were conducted using Python 3.10 and PyTorch 2.0.

### 2.4. Model Development and Validation

Using the preprocessed dataset, we evaluated the CNNs using a four-fold cross-validation approach. The CNNs were optimised using the Adam optimiser to minimise the binary cross-entropy loss [7,8]. Hyperparameters, such as the learning rate and mini-batch size, were selected based on preliminary experiments. To address class imbalance, a weighted loss computation was employed, multiplying each class weight by its respective loss value. Specifically, class weights were set at a 1:2 ratio for pEF relative to (mrEF + rEF). During training, except for the reduced lead ECG analyses, we applied the MixUp technique to enhance model performance [9].

For each cross-validation fold, the model checkpoint with the best performance on the development dataset was selected. This model was then assessed on a unified test fold (internal validation), which had been previously held out.

The models’ performance was assessed at the case level using both internal and external validation data, except for analyses involving reduced ECG leads, which employed data-level assessment. Evaluation metrics included the area under the receiver operating characteristic curve (AUC), accuracy, sensitivity, and specificity. AUC values served as the primary evaluation metric, whereas accuracy, sensitivity, and specificity were secondary.

The contribution of each lead was defined as the difference between the average metrics of models, including the specified lead and those excluding it.

Continuous data were presented as mean ± standard deviation for normally distributed variables. Categorical data were presented as numbers and percentages. Non-normally distributed data were presented as median values (lower-upper quartiles). Statistical analyses included the chi-square, Kruskal–Wallis, Student’s *t*-test, and Tukey-HSD tests combined with a one-way analysis of variance, as appropriate. Statistical significance for global test statistics was set at 5%. Analyses were conducted using JMP Pro software version 17 (SAS Institute, Cary, NC, USA) and custom Python scripts on MacOS computers.

## 3. Results

### 3.1. Comparison of 3 s ECG, Single-Beat ECG, and Two-Beat ECG Models for Detecting Ventricular Dysfunction Subsection

**Figure 4:**
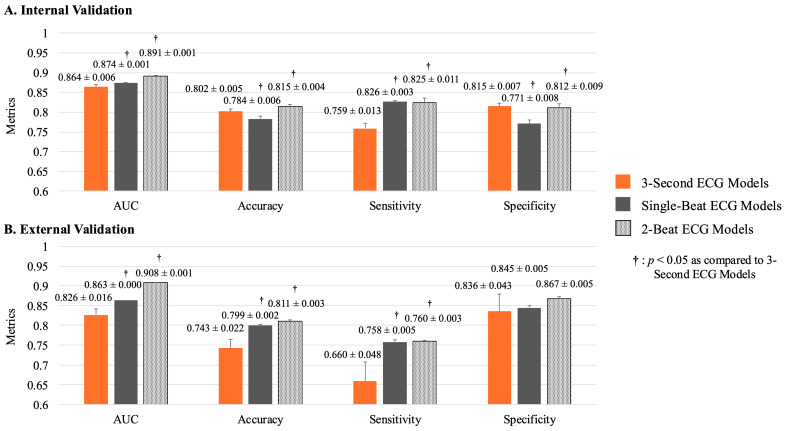
Performance of 3 s ECG and beat-wise ECG models. Performance comparisons of the 3 s ECG, single-beat ECG, and 2-beat ECG models are presented. (**A**) During internal validation, the two-beat ECG models outperformed the other two models. The single-beat ECG models exhibited superior performance regarding AUC and sensitivity, whereas the 3 s ECG models demonstrated superior accuracy and specificity. (**B**) During external validation, the two-beat ECG models again outperformed the other two models. The single-beat ECG models showed superior performance compared to the 3 s ECG models. ECG, electrocardiogram; AUC, area under the curve.

The AI models developed using 12-lead, 3 s ECG data successfully detected ventricular dysfunction with high accuracy, achieving an AUC of 0.864 ± 0.006 during internal validation. In contrast, models constructed from 12-lead, single-beat ECG data demonstrated significantly improved performance, with an AUC of 0.874 ± 0.001 compared to 0.864 ± 0.006 for the 3 s ECG models (*p* = 0.0095) (Figure 4). Models based on two-beat ECG data exhibited superior performance to the 3 s and single-beat models, with AUC values of 0.891 ± 0.001, 0.864 ± 0.006, and 0.874 ± 0.001, respectively (*p* < 0.0001).

During external validation, the single-beat ECG model outperformed the 3 s ECG model, achieving a superior AUC of 0.863 ± 0.001 versus 0.826 ± 0.016 (*p* = 0.0008) (Figure 3). The two-beat ECG model demonstrated further superior performance compared to the 3 s ECG and single-beat ECG models, with AUC values of 0.908 ± 0.001, 0.826 ± 0.016 and 0.863 ± 0.001, respectively (*p* < 0.0001).

### 3.2. Effect of Lead Reduction and Lead Configuration

The models were developed for each of the 40,955 possible combinations of the 12 leads in each cross-validation fold, undergoing both internal and external validations. For this analysis, the performance of the models was assessed at the data level rather than at the case level.

**Figure 5:**
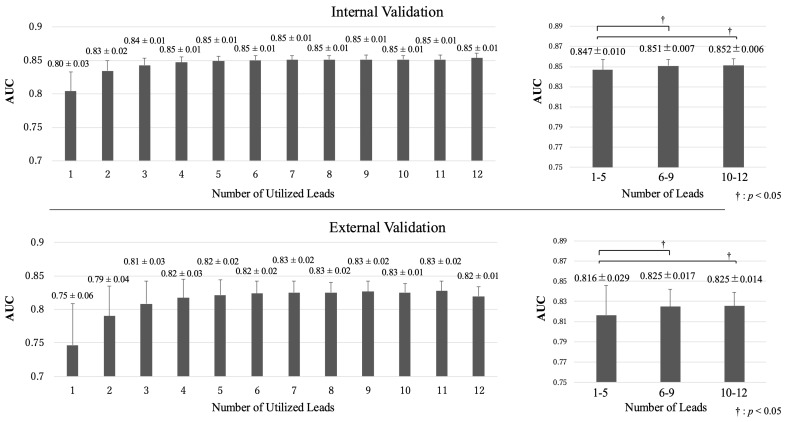
The number of utilised leads and the performance of models. Assessments of the models’ performance with a reduced number of leads are shown. During internal validation, the models constructed from the 12-lead ECGs showed the best performance. When categorising the number of leads into three groups—1–5 leads, 6–9 leads, and 10–12 leads—the models using 1–5 leads showed lower performance than those using 6–9 and 10–12 leads. During external validation, the models using 11 leads performed the best; however, the standard deviations were larger than those in the internal validation. Using the same three categorised groups based on the number of leads, the models using 1–5 leads again showed lower performance than those using 6–9 and 10–12 leads. Only the values in the left panels are shown with two significant figures for better readability. ECGs, electrocardiograms.

Initially, reducing the number of leads from 12 had a minimal impact on accuracy. However, a significant decline in the diagnostic accuracy was observed when the number of leads was reduced to five or fewer (Figure 5). Models with five or fewer leads showed an AUC of 0.847 ± 0.010, which was significantly lower than that of models with 6–9 leads or 10–12 leads (0.851 ± 0.007, 0.852 ± 0.006, *p* < 0.05, respectively) during the internal validation. In the external validation, the results were similar, with models having five or fewer leads exhibiting lower performance as compared to those with six or more leads (AUC 0.816 ± 0.029 versus 0.825 ± 0.017, 0.825 ± 0.014, *p* < 0.05, respectively).

**Figure 6:**
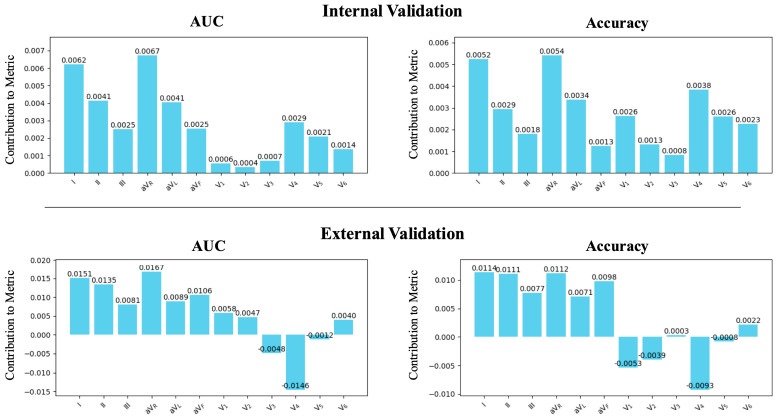
Contribution of each single lead. The contributions of each lead were calculated as the difference between the average metrics of the models that included the specified lead and the average metrics of the models that did not include the lead. During internal validation, the contribution of the limb leads was generally larger compared to the precordial leads. In external validation, the precordial leads showed a negative contribution to the detection of ventricular dysfunction.

Upon examining the contribution of each lead, the limb leads, especially leads I and aVR, were associated with an enhanced detection performance of the AI in both internal and external validations (Figure 6). Although all leads contributed to enhancing model performance in the internal validation, results from the external validation indicated that only the limb leads improved the models’ performance.

### 3.3. Comparison of Partial ECG Models

**Figure 7:**
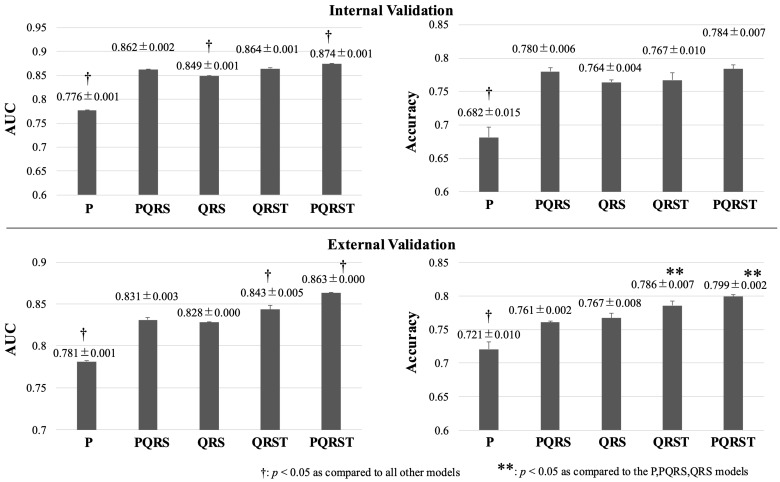
Performance of ECG segment models. Comparisons of performance among the ECG segment models are shown. In both internal and external validation, the PQRST model significantly outperformed the other models. The P segment and QRS segment models exhibited inferior performance compared to the PQRS, QRST, and PQRST models. ECG, electrocardiogram.

In the internal validation, the models built with the PQRST (⑤) segment, representing the entire single-beat ECG, demonstrated the highest performance (AUC 0.874 ± 0.001) (Figure 7). The PQRS (②) and QRST (④) models exhibited the next highest performance, with no significant difference between them (AUC 0.862 ± 0.002 and 0.864 ± 0.001, respectively). The P (①) segment model performed the poorest, and the QRS (③) model was only slightly more accurate (AUC 0.776 ± 0.001 and 0.849 ± 0.001, respectively).

In the external validation (Figure 6), the PQRST (⑤) model exhibited the highest accuracy, followed by the QRST (④) model (AUC 0.863 ± 0.001 and 0.843 ± 0.005, respectively). The performances of the other three models were significantly lower than these two models (AUC 0.781 ± 0.001, 0.831 ± 0.003, and 0.828 ± 0.004, for P (①), PQRS (②), and QRS (③) segment models, respectively, *p* < 0.05).

## 4. Discussion

The main findings of this study were as follows: (1) AI models developed using single-beat ECG and two-beat ECG data demonstrated higher performance in detecting ventricular dysfunction compared to those constructed from 3 s ECG data; (2) increasing the number of beats improved the performance of the models; (3) the diagnostic capability of AI was maintained when the number of leads was reduced from 12 to 6; and (4) both the QRS complex and ST-T segments were crucial for detecting ventricular dysfunction from an ECG.

Traditionally, deep learning models utilise ECG data in the form of strips containing multiple heartbeats, encompassing both the systolic and diastolic phases of the cardiac cycle [10,11]. One of the key features of this study was the adoption of beat-wise ECG analysis and its comparison with traditional strip-type ECG analysis models. Using the beat-wise approach, we segmented the ECGs into distinct intervals and evaluated the accuracy of recognising ventricular dysfunction. To the best of our knowledge, this has not been previously documented. Previous reports on beat-wise ECG analyses have mainly focused on rhythm classification [12,13,14].

The high accuracy of the AI models developed from the single-beat ECG data, as evidenced by an AUC value exceeding 0.85, suggests that a single heartbeat waveform contained adequate information for diagnosing ventricular dysfunction. Additionally, our results demonstrated that leveraging multiple heartbeats of the cardiac cycle improved the performance of these models. However, our findings did not conclusively determine the importance of ECG segments between the end of the T-wave and the subsequent PQRST complex, such as the U-wave. Further research is warranted to elucidate the significance of these ECG intervals and their potential impact on diagnostic accuracy.

The maintained AI performance, even with the reduction to as few as six leads, suggested that each lead contributed to the detection of ventricular dysfunction. However, lead-specific analysis revealed that limb leads, particularly aVR and I, contributed significantly more to the detection of ventricular dysfunction. This variability in contribution among leads underscores the importance of selecting optimal leads for diagnostics. Although some publications have highlighted aVR’s importance, clinical practice finds both aVR and I offer limited information [15,16].

A previous study demonstrated that the majority of ECG interpreters, even those with at least one year of experience, tend to overlook the information provided by lead aVR [17]. The authors of that study suggested that this neglect may be due to the fact that lead aVR is unique in presenting a negative deflection, while the other eleven leads display positive deflections towards either the left or right ventricular chambers. This difference often results in interpreters disregarding lead aVR during clinical assessments. However, contrary to this tendency, Selvester et al. highlighted that lead aVR can provide valuable information for estimating myocardial infarct size [18]. This may be due to the fact that lead aVR is thought to reflect the endocardial surface of the left ventricle [19], which is a focus of the present study.

ECGs resembling lead I signals can be recorded using commercially available wearable devices, such as smartwatches [20]. A previous study demonstrated that ECGs recorded using wearables can provide sufficient diagnostic performance for patient screening [21].

In segment analyses, the significance of the QRS segment for detecting the ventricular ejection fraction was expected, given its representation of ventricular contraction crucial for diagnosing ventricular dysfunction. However, models relying solely on QRS segments performed poorly. The inclusion of additional information from the ST segment and the T-wave, as well as the P segment, improved model accuracy. This highlights the importance of a comprehensive analysis of the entire PQRST complex in enhancing diagnostic capabilities for ventricular dysfunction. Given the diverse range of ECG waveforms, visually distinguishing between normal and abnormal conditions remains challenging. Continued research in this area is expected to identify unknown signals of cardiac dysfunction, potentially leading to significant advancements in diagnostic processes.

Typically, an ECG comprises eight original waveform leads (I, II, and V1–V6) and four derived leads (III, aVR, aVL, and aVF), measured from the limbs and chest. The greater utility of limb leads, particularly I and aVR, in detecting ventricular dysfunction in this study suggests that they may be more informative than precordial leads, especially in diverse populations. This inference is supported by the diminished contribution of precordial leads in external validation, which may reflect differences in physique between Japanese and German individuals [22,23]. The extent to which physique affects ECG waveforms warrants further investigation.

This study had several limitations. Firstly, the AI models were developed based on an ECG database from a single centre, which potentially affected the generalisability of the findings. To address this, external validation was conducted to confirm the generalisability of the constructed models. Secondly, the partial ECG segments, defined as the ‘P’ segment, included data from patients with atrial fibrillation (AF) who lacked a P-wave in these segments. In this context, ‘P’ refers to the time point preceding the QRS segments. Thirdly, we adopted the German ECG dataset for external validation, although model development was conducted using the Japanese ECG dataset. Variations in signals indicating ventricular dysfunction may exist among different ethnic groups. However, the results of external validation showed that the Japanese ECG models performed effectively on the German dataset. In contrast, the characteristics of precordial leads might have differed between the two populations. Fourthly, our models did not provide information about the aetiology of left ventricular dysfunction, such as ischaemic versus non-ischaemic origin, even though these conditions influence ECG patterns. Additionally, our analysis focused on reduced left ventricular ejection fraction, and the presence and severity of comorbid valvular diseases were not explicitly considered. Finally, the CNN architectures used in this study were based on previous studies and preliminary analyses (Supplemental Figure S1) [24,25,26]. This suggests that exploring other novel architectures may improve performance. Despite these limitations, we believe that our results can be generalised, as other prevalent architectures, such as ResNet or Inception, were also tested during the preparatory phase of this study. These tests yielded similar or even inferior detection performances compared to the architectures adopted in this study.

## 5. Conclusions

The indicators of ventricular dysfunction on the ECG were primarily located between the QRS- and T-waves, with the P segment exhibiting some, albeit lower, significance. Using multiple heartbeat waveforms significantly enhanced the performance of the models, outperforming those based on ECG strips. This demonstrates that beat-wise ECGs are more effective in capturing signals of ventricular dysfunction than ECG strips spanning 3 s, offering a more focused and potentially more precise diagnostic tool.

## Figures and Tables

**Table 1:** Research participants.

Institution	Number of Unique Cases	Number of ECGs	LVEF	For Train and Dev	For Test
JMU	10,626	17,021	pEF	10,398	2677
			mrEF	1452	387
			rEF	1709	398
UHD	401	401	pEF		190
			mrEF		159
			rEF		52

ECG, electrocardiogram; JMU, Jichi Medical University; LVEF, left ventricular ejection fraction; UHD, University Hospital Düsseldorf; pEF, preserved ejection fraction; mrEF, mildly reduced ejection fraction; rEF, reduced ejection fraction.

## Data Availability

The patient data underlying this article cannot be shared publicly to protect the privacy of the individuals who participated in the study. The data regarding the AI models will be shared upon reasonable request to the corresponding author.

## Supplementary Materials

The following supporting information can be downloaded at: https://www.mdpi.com/article/10.3390/bioengineering11111069/s1, Figure S1: Performance of Models during Preliminary Experiments.
