# Supplementary material for: Identifying Ventricular Dysfunction Indicators in Electrocardiograms via Artificial Intelligence-Driven Analysis
Source: Bioengineering (Basel). 2024 Oct 26;11(11):1069. doi: 10.3390/bioengineering11111069 (PMC11591176; doi:10.3390/bioengineering11111069)
Supplement: Supplementary file 1 [file bioengineering-11-01069-s001.zip › bioengineering-3237487-supplementary.pdf]

Supplemental Material for „Identifying ventricular dysfunction indicators in electrocardi-ograms via artificial intelligence-driven analysis“

Hisaki Makimoto 1,2,3,4,*, Takayuki Okatani 2, Masanori Suganuma 2, Tomoyuki Kabutoya 1, Takahide Kohro 1,3, Yukiko Agata 1, Yukiyo Ogata 1, Kenji Harada 1, Redi Llubani 4, Alexandru Bejinariu 4, Obaida R Rana 4, Asuka Makimoto 4,5, Elisabetha Gharib 4, Anita Meissner 4, Malte Kelm 4,6 and Kazuomi Kario 1

1. Cardiovascular Centre, Jichi Medical University, Shimotsuke, Japan
2. Graduate School of Information Sciences, Tohoku University, Sendai, Japan
3. Data Science Centre, Jichi Medical University, Shimotsuke, Japan
4. Division of Cardiology, Pulmonology and Vascular Medicine, Medical Faculty, Heinrich-Heine University, Düsseldorf, Germany
5. Clinical Research Center, Jichi Medical University, Shimotsuke, Japan
6. CARID, Cardiovascular Research Institute Düsseldorf, Medical Faculty and University Hospital Düsseldorf, Heinrich-Heine-University Düsseldorf, Germany

Modified Stratified Four-fold Cross Validation

We adopted the four-fold cross-validation approach based on the following considerations.

We intended to conduct an internal test using a fixed, predefined cohort with the same sample size as the development cohort, i.e. validation cohort during training. To achieve this, we divided the Jichi sample into five groups, ensuring that the proportions of patients with preserved ejection fraction (pEF), mildly reduced ejection fraction (mrEF), and reduced ejection fraction (rEF) were consistent across all groups. We also assured that there were no overlapping cases across the groups.

One of these groups was arbitrarily designated as the internal test cohort, while the remaining four groups were used for training in a four-fold cross-validation framework. This approach allowed us to maximize the use of the data for model training and validation while maintaining an internal test set with a representative patient distribution.

Furthermore, our research team has previously employed a similar four-fold cross-validation method in an earlier study, yielding optimal results [25, 26]. This prior experience informed and supported our decision to use this method in the present study.

Structure of CNN Model

Our CNN model is designed for 1D signal processing of ECG data from 12 channels (12-lead ECG). The model extracts features from the input through multiple layers of 1D convolution and pooling operations, progressively refining and transforming the input into meaningful representations for classification.

1. Input Structure and Shape

The model’s input has the shape [batch_size, 12, sequence_length], where 12 represents the number of input channels (12-lead ECG), and sequence_length corresponds to the number of time steps in each signal. In the present study:

- 3-second ECG had a sequence_length of 1500 (3 seconds * 500 Hz).
- one-beat ECG and segment ECG (except atrial parts) had a sequence_length of 375.
- segment ECG for atrial parts had a sequence_length of 100.

Each channel corresponds to an individual ECG lead, and the model learns to extract patterns from both single and multi-channel inputs.

2. Convolutional Layers and Kernels

The core operation in each convolutional layer is defined by kernels (filters). A kernel in 1D convolution slides over the input signal along the time axis, detecting patterns across consecutive time steps.

- Kernels: In the first convolutional layer (Conv1), the kernel size is set to 5. This means the filter considers 5 consecutive time points (equivalent to 10 ms) from the input data as it moves along the sequence.
- Input Channels (12 Leads): Each kernel in Conv1 operates across all 12 input channels (ECG leads) simultaneously, allowing it to capture interactions between different leads within the 5 time-step window. In Conv1, there are 16 kernels, each learning to extract distinct features from the input.
- Subsequent Layers: As the data progresses deeper layers to Conv3, Conv4, and beyond, the kernel size decreases (shrinking to 3), and the number of output feature maps increases (from 16 in Conv1 to 256 in Conv10). These layers refine the learned features by building on the feature maps produced by the earlier layers.

3. Temporal and Multi-Channel Feature Extraction

- Temporal Features: The kernel size determines the temporal scope of the input that ´ is captured by each convolution operation. A kernel size of 5 in the first layers enables the model to capture localized temporal features, such as spikes, dips, or oscillations that are typical in ECG.
- Multi-Channel Processing: Since each kernel spans all 12 input channels, the model learns to detect complex patterns across multiple ECG leads, which is crucial for understanding lead-to-lead interactions.

4. Pooling Layers

After each convolutional blocks, the model applies a max pooling operation (MaxPool1D), which reduces the size of the feature maps along the time dimension. Max pooling retains the most significant activation within a defined window, helping the model focus on the most relevant features and reducing computational load. Pooling also downsamples the data, preserving important patterns while discarding less relevant details. In the present study, we a pooling kernel size of 2 was used, reducing the signal size by half at each pooling layer.

5. Global Pooling and Fully Connected Layer

Following multiple convolutional and pooling layers, the model applies global average pooling (AdaptiveAvgPool1D), effectively eliminating a time dimension. This results in a fixed-size representation of the input data, which then passed to a fully connected layer (Linear) that maps the

features to a 2-dimensional output for binary classification.

6. Feature Hierarchy and Abstraction

In general, in the earlier layers, such as Conv1, the model learns simple, local features such as sharp spikes, dips, or waveforms that occur over short time intervals across the 12 ECG channels. As the data progresses through deeper layers (Conv5 to Conv10), the model captures more abstract and complex features by combining information from earlier layers. These high-order features may correspond to broader patterns or diagnostic signals, which are essential for accurate classification.

Preliminary Experiments

As shown on Table 1, our clinical data was imbalanced. In the preliminary analysis using 3-second ECG data, we evaluated the the following conditions: 1) imbalanced data, 2) balanced data using class-weight, 3) MixUp, and 4) MixUp with balanced data using class-weight.

Supplemental Figure 1 presents the performance of the models under each condition. From the perspective of area under the receiver operator characteristics curve (AUROC), there were no significant differences among the four conditions.

Models using class-weighted balanced data exhibited higher sensitivity; however, accuracy, F1 score, and specificity were reduced. In contrast, models incorporating MixUp demonstrated improved accuracy and specificity but lower F1 scores and sensitivity. Models combining both class-weight and MixUp achieved higher sensitivity, a comparable F1 score to the imbalanced data, but lower accuracy and specificity.

Specifically, in the detection of reduced ejection fraction (LVEF < 40%), as shown in panel F of Supplemental Figure 1, the incorporation of class-weight improved the identification of severe LV dysfunction.

Based on these results, particularly the preserved F1 score and enhanced detection of reduced ejection fraction, we decided to employ both class-weight and MixUp in the present study.

Supplemental Figure S1. Performance of Models during Preliminary Experiments

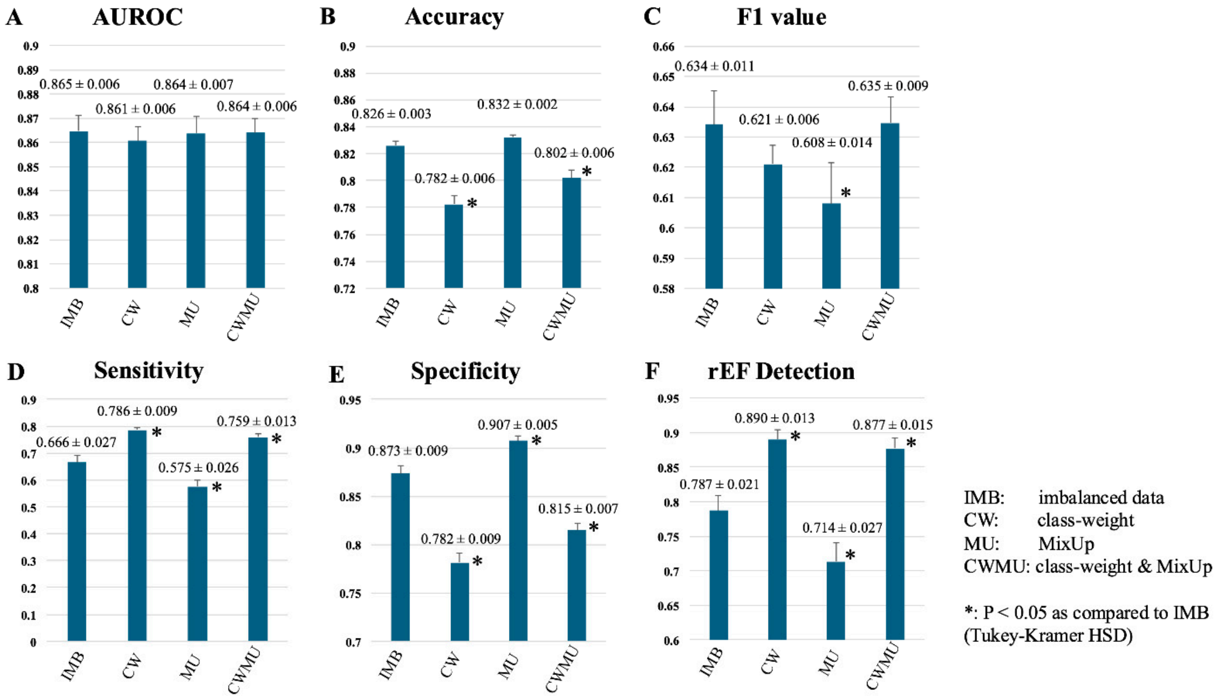

The performance of the models trained by imbalanced data, balanced data using class-weight, imbalanced data with MixUp, and balanced data using class-weight with MixUp are shown. See text for details.
